# Misinformation in Wake of the COVID-19 Outbreak: Fueling Shortage and Misuse of Lifesaving Drugs in Pakistan

**DOI:** 10.1017/dmp.2020.400

**Published:** 2020-10-22

**Authors:** Furqan Khurshid Hashmi, Naveel Atif, Usman Rashid Malik, Zineb Riboua, Fahad Saleem, Yusra Habib Khan, Abrar Ahmad, Tauqeer Hussain Mallhi

**Affiliations:** University College of Pharmacy, University of the Punjab, Allama Iqbal Campus, Lahore, Pakistan; Center for Drug Safety and Policy Research, School of Pharmacy, Xi’an Jiaotong University, China; McCourt School of Public Policy, Georgetown University, Washington, DC; Faculty of Pharmacy, University of Balochistan, Quetta, Pakistan; Department of Clinical Pharmacy, College of Pharmacy, Jouf University, Sakaka, Kingdom of Saudi Arabia; Faculty of Pharmacy, University of Central Punjab, Lahore, Pakistan

**Keywords:** COVID-19, life saving drugs, misinformation, precautionary measures

The 2019 coronavirus disease (COVID-19), declared a pandemic by the World Health Organization (WHO), is spreading worldwide rapidly.^1^ As of August 9, 2020, the total death toll had reached about 0.72 million with approximately 19.4 million confirmed cases worldwide.^2^ The WHO stated that “Our greatest concern is the potential for the virus to spread to countries with weaker health systems, and which are ill-prepared to deal with it.”^3^ In Pakistan, a lower middle-income country, the outbreak of COVID-19 constitutes a significant challenge: a struggling economy with limited resources, inadequate health care system, and infrastructure. The role of the Government of Pakistan in the dissemination of COVID-19-related information through various media sources is appreciable. However, a clamoring issue is to deal with rumors and wide-spread misinformation and disinformation regarding COVID-19 treatment.^4^

Misinformation, disinformation, and rumors regarding COVID-19 treatments are responsible for creating mistrust among the general public, thus leading to self-medication, panic-buying, and misuse of drugs. The prophylactic use of chloroquine/hydroxychloroquine (alone or in combination with azithromycin), methylprednisolone, tocilizumab, ivermectin, and remdesivir for COVID-19 is escalating throughout the world.^5,6^ This spread of misinformation is rampant and has strengthened the false beliefs of laymen and non-specialists after the misinterpreted and distorted health care specialists’ statements regarding the use of abovementioned drugs as a COVID-19 cure. As a result, many people, including the general public and marketers in Pakistan, have panic-bought and hoarded these drugs, causing a shortage of these lifesaving medicines in the market. Preliminary evidence has reported that hydroxychloroquine shows antiviral activity in-vitro against coronaviruses, particularly in severe acute respiratory syndrome coronavirus 2 (SARS-CoV-2).^7^ The adverse effects associated with the use of antimalarial drugs like retinopathy and neurotoxicity should not be undermined, particularly chloroquine, which tends to cause torsades de pointes associated with QT interval prolongation and has the potential to cause life-threatening ventricular tachyarrhythmia in cardiac patients.^8^ Preliminary results of a study in China found that methylprednisolone diminishes the mortality in patients with severe COVID-19; however, almost half of the patients died (23/50). Therefore, the use of methylprednisolone warrants further investigation.^9^ A systematic review of studies recommends the use of tocilizumab as a compassionate option on COVID-19 patients but also warns to perform latent tuberculosis testing during and before the therapy as it may reduce the antimicrobial activity of IL-6.^10^

For public safety concerns associated with self-medication, health authorities in Pakistan must use social media tracking tools and SMS services to dispel any kind of misinformation or disinformation regarding the misuse of aforementioned drugs. The current shortage of these drugs can be encountered by using a multipronged approach, that is, by enhancing the production, improving the drug supply chain, and by taking all stakeholders, including marketers on board, to address this pressing issue. A long-term measure to address the shortage of lifesaving drugs is by restricting the non-prescription medicines dispensing, and by enforcing prescription-only medicines dispensing across the country to avoid any shortage and crisis. Pharmacists, being on the front line during the current crises, may assist to curb the situation by providing education to the general community as well as clinicians. We urge health authorities to ascertain the pattern of drug use in the general community by involving community pharmacists so that timely measures could be taken.^11^
